# Clinical characteristics associated with increased resource utilization of hospitalized children under 5 years with acute gastroenteritis at a tertiary hospital in the northern region of Ghana: a retrospective study

**DOI:** 10.11604/pamj.2019.33.186.13133

**Published:** 2019-07-11

**Authors:** Alhassan Abdul-Mumin, Sean Ervin, Elizabeth Eby Halvorson

**Affiliations:** 1 Department of Pediatrics and Child Health, University for Development Studies, School of Medicine and Health Sciences and Tamale Teaching Hospital, Tamale, Northern Region, Ghana; 2 Department of Pediatrics, Wake Forest Baptist Medical Center, Medical Center Boulevard, Winston Salem, NC 27157 USA

**Keywords:** Acute gastroenteritis, low resource setting, resource utilization, length of stay

## Abstract

**Introduction:**

Acute gastroenteritis (AGE) is a leading cause of mortality in children in developing countries. Management of AGE consumes medical resources, including antibiotics and intra-venous fluids, but factors affecting resource utilization in the management of AGE are under-studied. We hope to identify clinical predictors of resource utilization in AGE.

**Methods:**

We performed a retrospective chart review of patients 1-60 months of age admitted to a tertiary hospital in Northern Ghana between January 2013 and December 2014 with an admitting diagnosis of AGE. We collected data on patient demographics, presenting symptoms, and subsequent management. Our primary outcome was prolonged hospital length of stay, defined as >4 days. Secondary outcomes included other measures of resource utilization, such as use of antibiotics, antimalarials and intravenous fluids. Demographic and clinical characteristics were compared between groups with Pearson chi square test for categorical variables and ANOVA for continuous variables. Multivariable logistic regression modeling for each outcome included all variables found to be significant in the bivariate analysis.

**Results:**

We reviewed charts for 473 patients admitted for AGE during this timeframe. 264 (56%) were male, median age was 12 months. 448 (95%) received antibiotics, 396 (84%) received antimalarials and 365 (77.2%) received intravenous fluids. 167 (35.3%) had prolonged LOS >4 days. Following multiple logistic regression analysis, clinical features associated with prolonged LOS included fever duration (OR 2.87, 95% CI 2.28-3.61 per 1-day increase), mild (OR 2.39, 95% CI 1.12-5.08) or moderate (OR 3.13, 95% CI 1.57-6.21) dehydration (compared to none) and symptom duration (OR 1.13, 95% CI 1.01-1.27 per 1-day increase).

**Conclusion:**

Dehydration and duration of symptoms prior to presentation predict prolonged hospital LOS in young children with AGE in Northern Ghana.

## Introduction

In developing countries acute gastroenteritis (AGE) is second only to pneumonia as a cause of mortality in the first 5 years of life, contributing to more than half a million deaths annually [1]. It is a major cause of morbidity in the developed world [2]. The management of AGE is based on clinical findings [3] with a focus on the correction of dehydration and optimizing fluid and nutritional support. The characteristics of the diarrheic stool as bloody, mucoid or watery may assist in determining those patients most likely to require supplemental antimicrobial therapies; however, management practice can vary between practitioners, institutions and countries [4, 5]. A study conducted in Tanzania found that 80% of children with acute watery diarrhea received antibiotics and that the frequency of prescription of antibiotics depended on the type of prescriber [6]. One of the reasons for the variations in management is the inconsistencies in guidelines and recommendations for managing AGE in children [7, 8]. Much of the variation in practice is related to the use of antibiotics in the management of AGE [9].

It is estimated that 70% of cases of AGE are caused by viral infection, with rotavirus being the most common worldwide. Data from southern Ghana support the high prevalence of rotavirus associated acute gastroenteritis [10]. In contrast, there is a relative gap of knowledge pertaining to the presentation of AGE and management strategies in the northern region of Ghana. More generally, as AGE may present with a concomitant fever, management is confounded by the multiple etiologies for febrile illness in this part of the world [11, 12]. The Ghana standard treatment guidelines (GSTG) outline the management of AGE in Ghana [13]. The GSTG describes the most common etiology of AGE as viral and cautions against the use of antibiotic in most cases. The practitioner is advised to document the characteristics of the stool as watery, mucoid or bloody; the presence of fever associated vomiting and reduced urine output. Degree of dehydration is estimated from the general clinical condition, appearance of the eyes, presence of tears, moisture content of the mouth and tongue, skin turgor and the presence of thirst. This allows the degree of dehydration to be graded as none to severe based on clinical documentation. Recommended routine investigations for severe AGE may include a full blood count (CBC), blood film for malarial parasites (BF), routine stool examination (R/E), stool for culture and sensitivity (C/S) and a measure of the blood urea and creatinine. Based on the findings above the treatment plan is based on fluid resuscitation either as an outpatient or as an inpatient. Diagnostic testing is aimed at deciding if the infection is most likely viral, bacterial, or protozoal. Specific recommendations are made for indications for antimicrobial therapy and supplemental zinc is recommended for all children.

Our study’s objective was to determine the clinical presentation and management of children under 5 years old presenting to the Tamale Teaching Hospital (TTH), a tertiary referral health facility in Northern Ghana with AGE. We hypothesized that many children would not receive the standard treatment therapy during episodes of acute diarrhea. We sought to understand whether inappropriate prescriptions for intravenous fluids, antimalarials or antibiotics as measures of resource utilization were prevalent during the inpatient management of AGE and if they correlated with length of stay. In addition, we reviewed the charts for documentation of care delivery, clinical assessment for dehydration, fever and stool characteristics and use of appropriate medications as zinc, oral rehydration solution (ORS) and when indicated, intravenous fluids (IVF). We hoped to identify predictors of increased resource utilization (length of stay, use of antimicrobials, appropriate institution of rehydration therapy with respect to degree of dehydration-e.g., oral followed by intravenous support only when intolerant of orals) for AGE.

## Methods

**Study site:** Tamale Teaching Hospital is the only tertiary referral health facility in the northern part of Ghana. The bed capacity of the hospital is about 450 with a pediatric bed capacity of 100. It is located in the Tamale metropolis of the northern region of Ghana and the population of the catchment area is about 2.4 million. About 350,000 of the population are children under 5 years.

**Study design:** we conducted a retrospective chart review of children 1-60 months who were admitted to the general pediatric ward of TTH with the diagnosis of AGE between January 2013 and December 2014.

**Study population selection:** all children aged 1-60 months who were admitted to the pediatric ward of the hospital with AGE were eligible for the study. Children with severe acute malnutrition were excluded from the study because of inherent difficulties in assessing dehydration and the differences in management for these patients.

**Definition of acute gastroenteritis** we adopted a standard case definition for acute gastroenteritis; “as an individual with ≥3 loose stools, or any vomiting in 24 hours and excluding those with chronic illness with symptoms of vomiting and diarrhea or those who report their symptoms are due to medications” [14]. Allied symptoms that may present with acute gastroenteritis include signs of dehydration, fever and blood in stools. Notes were made of these when documented in the clinical record.

**Outcome measures:** our primary outcome was prolonged LOS, defined as greater than the median of 4 days. There is not a robust literature on the expected LOS for children admitted with this condition in West Africa; however one study from the upper east region of Ghana (Navrongo) [15] reported an average length of stay of 3.5 days for hospital treatment for AGE with rehydration, antibiotics or antimalarials. Therefore, we developed this definition for prolonged LOS of >4 days post hoc from our own cohort and by reference to this study. Secondary outcomes included other measures of resource utilization; any use of antibiotics, number of antibiotics administered, any use of antimalarials and any use of intravenous fluids.

**Data collection:** we reviewed the hospital ledger for subjects who met our inclusion and exclusion criteria during the study period then identified available patient folders for chart review. We collected age of patient, sex, presenting complaint(s) and duration of complaint. Physical examination findings recorded include temperature at presentation, general appearance of the patient and eagerness to drink, skin recoil time, sunken eyes and degree of dehydration. We also investigated the frequency and documentation of laboratory investigations, including stool R/E, stool C/S, FBC, blood film (BF) for malaria parasites and C/S. We collected information on the use of ORS, IVF, antimalarials, antibiotics and zinc supplementation. We recorded duration of fever and LOS in the hospital.

**Data analysis:** statistical analyses were performed using SAS version 9.4 (SAS Institute Inc. Cary, NC). Outcomes considered were prolonged LOS and use of antimalarial, antibiotics, and IVF. Descriptive statistics of admission characteristics were compared against these outcomes using Pearson chi square analysis for categorical variables and ANOVA for continuous variables. Many of our variables were not normally distributed; these results are presented as medians with interquartile ranges (IQR). Univariate analyses were performed to identify factors associated with our outcomes, including age, sex, presenting symptoms and duration of symptoms. Factors found to be significantly associated in the univariate analysis (with a p<0.01) were included in multivariable logistic regression modeling. A p value of <0.05 was used to define statistical significance for all other testing.

**Ethical considerations:** ethical clearance for this study was obtained from the ethical review board of the TTH (Approval no: TTH/24/10/15/02). All relevant data were retrieved from available patient folders and there was no contact with study participants. All patient identifiers were de-identified in the final analyses.

## Results

A total of 473 patients meeting our inclusion and exclusion criteria were included in the study. The median age of patients in this cohort was 12 months (IQR 9-24), and 264 (56%) were male. The most common presenting complaint was both diarrhea and vomiting (356 patients, 75%), with the remainder of our cohort reporting either one or the other. Stool was most commonly reported to be watery (267 patients, 56%). All patients had been assessed for dehydration. 344 (75%) had no dehydration, 45 (8%) had mild, 65 (13%) had moderate, and 52 (4%) had severe dehydration at presentation. Our primary outcome, hospital LOS, ranged from 1-16 days, with a median of 4 days (IQR 3-5). Therefore, for our study, prolonged LOS was defined as >4 days. Patient demographics and clinical characteristics are categorized by those with short versus prolonged LOS in Table 1. In the univariate analysis shown, young age, degree of dehydration and fever presence and duration were all associated with prolonged LOS.

**Table 1:** Demographics and clinical features at presentation by LOS

	LOS<=4 days (N=306) N (%)	LOS>4 days (N=167) N(%)	P value
**AGE**			
1-12 months	88 (29%)	72 (43%)	
12-24 months	107 (35%)	59 (35%)	<0.001
24-59 months	111 (36%)	36 (22%)	
**MALE SEX**	171 (56%)	93 (56%)	0.91
**SYMPTOM DURATION**			
<2 days	102 (33%)	39 (23%)	0.02
>= 2 days	204 (67%)	128 (77%)	
**PRESENTING SYMPTOM(S)**			
Diarrhea alone	63 (21%)	29 (17%)	
Diarrhea/vomiting	226 (74%)	130 (78%)	0.63
Vomiting alone	17 (6%)	8 (5%)	
**STOOL CHARACTERISTICS**			
Bloody	9 (3%)	2 (1%)	
Watery	165 (54%)	102 (61%)	0.32
Mucoid	95 (31%)	48 (29%)	
N/A	37 (12%)	15 (9%)	
**DEGREE OF DEHYDRATION**			
None	241 (79%)	103 (62%)	
Mild	25 (8%)	20 (12%)	<0.001
Moderate	33 (11%)	32 (19%)	
Severe	7 (2%)	12 (7%)	
**FEVER >38.6 AT PRESENTATION**	49 (16%)	43 (26%)	0.01
**FEVER DURATION**			
<=1 day	257 (84%)	50 (30%)	<0.001
>1 day	49 (16%)	117 (70%)	

**Diagnostic and treatment interventions:** frequencies of diagnostic and treatment interventions are included in Table 2. The most common laboratory investigations carried out were BF (401 patients, 85%) and CBC (291 patients, 62%). Stool examination, stool culture and blood culture were performed rarely in our cohort. All treatment interventions, including ORS, zinc, antibiotics, antimalarials, and IVF were used commonly in these patients (Table 2).

**Table 2:** Proportion of total cohort receiving diagnostic and treatment modalities

	N=473N (%)
**DIAGNOSTIC TESTING**	
Malarial blood film	401 (85%)
Complete blood count	291 (62%)
Stool examination	30 (6%)
Stool culture	10 (2%)
Blood culture	4 (1%)
**ORS**	343 (73%)
**ZINC TABLET**	397 (84%)
**ANTIBIOTICS**	
None	25 (5%)
1	109 (23%)
2	207 (44%)
≥3	132 (28%)
**ANTIMALARIAL**	396 (84%)
**IVF**	365 (77%)

**Figure 1:**
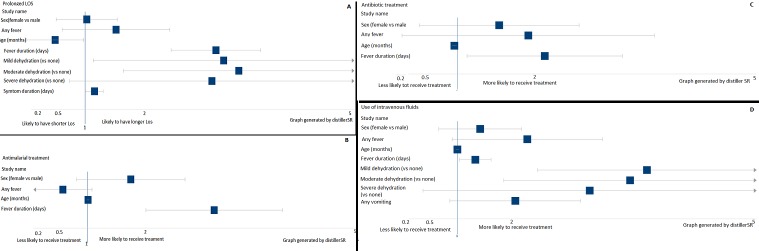
Associations between patient characteristics and measures of resource utilization following multivariable logistic regression modeling. a) Prolonged length of stay >4 days; b) Antimalarial treatment; c) Antibiotic treatment; d) Use of intravenous fluids

**Predictors of resource utilization:** we used multivariable logistic regression to identify demographic and clinical variables associated with prolonged LOS, antimalarial treatment, antibiotic treatment, and use of IVF (Figure 1, Table 3). Our primary outcome, prolonged LOS, was positively associated with fever duration (OR 2.87, 95% CI 2.28-3.61 per 1-day increase), mild (OR 2.39, 95% CI 1.12-5.08) or moderate (OR 3.13, 95% CI 1.57-6.21) dehydration (versus none), and symptom duration (OR 1.13, 95% CI 1.01-1.27 per 1-day increase). We found that fever duration was also associated with our other resource utilization outcomes, including antimalarial treatment, antibiotic treatment and use of IVF. Degree of dehydration was only associated with prolonged LOS and use of IVF. Age, sex and other presenting symptoms (fever, vomiting and diarrhea) were not associated with any outcomes studied.

**Table 3:** Associations between patient characteristics and measures of resource utilization following multivariable logistic regression analyses

	Odds Ratio (95% CI)
**Antimalarial treatment**	
**Sex**	
Male	Ref
Female	1.43 (0.84-2.45)
**Any fever**	
No	Ref
Yes	0.31 (0.09-1.07)
Age (per 1-month increase)	1.01 (0.99-1.03)
**Fever duration (per 1-day increase)**	**2.70 (1.87-3.90)**
**Antibiotic treatment**	
**Sex**	
Male	Ref
Female	1.13 (0.49-2.63)
**Any fever**	
No	Ref
Yes	0.97 (0.26-3.64)
Age (per 1-month increase)	0.96 (0.93-0.99)
Fever duration (per 1-day increase)	1.91 (1.13-3.21)
Use of IVF	
**Sex**	
Male	Ref
Female	1.18 (0.75-1.86)
**Any fever**	
No	Ref
Yes	1.65 (0.93-2.94)
**Degree of dehydration**	
None	Ref
Mild	8.74 (2.07-36.93)
Moderate	4.18 (1.62-10.84)
Severe	2.42 (0.54-10.97)
**Any vomiting**	
No	Ref
Yes	1.55 (0.90-2.65)
**Any diarrhea**	
No	Ref
Yes	1.19 (0.41-3.47)
Age (per 1-month increase)	1.00 (0.98-1.02)
Fever duration (per 1-day increase)	1.22 (1.03-1.45)
**Prolonged LOS**	
**Sex**	
Male	Ref
Female	0.92 (0.57-1.48)
Any fever	
No	Ref
Yes	1.22 (0.66-2.26)
**Degree of dehydration**	
None	Ref
Mild	2.39 (1.12-5.08)
Moderate	3.13 (1.57-6.21)
Severe	3.33 (0.77-14.5)
Symptom duration (per 1-day increase)	1.13 (1.01-1.27)
Age (per 1-month increase)	0.98 (0.96-1.00)
Fever duration (per 1-day increase)	2.87 (2.28-3.61)

## Discussion

AGE is a common pediatric illness and may have significant mortality, as high as 20% in some developing nations [1, 16]. There is dearth of reports in the literature describing the management of children aged 1-60 months admitted for AGE in Northern Ghana and especially assessing factors contributing to resource utilization in this part of the country [15]. Identifying these predictors may help clinicians to focus testing and treatment appropriately, especially in resource limited settings. Fever duration was associated with all resource utilization outcomes in this study, suggesting that prolonged fever may drive treatment for presumptive malarial and bacterial illness. Given the limited diagnostic testing performed in our cohort, we do not know the true prevalence of these disease entities. In one study in Southern Ghana rotavirus gastroenteritis accounted for up to 50% of cases admitted to hospital [10] and overall admissions for gastroenteritis range from 10-20% of common presenting illness requiring hospitalization in other parts of Africa [17, 18]. Fever may co-present with diarrhea and vomiting in acute gastroenteritis but is not invariably present. Only 19% of our patients admitted with AGE had a documented fever of >38.6^o^C. By both univariate and multivariate analyses, presence of fever did not statistically correlate with any variable measured; however fever duration was significantly correlated with the use of antibiotic, antimalarial and with LOS (Figure 1). The presence of fever complicates clinical decision making and management strategies in this setting. The management of acute gastroenteritis in our setting is likely complicated by the endemicity of malaria in this region and the protean manifestations of acute malarial illness including gastrointestinal symptoms and fever. This compels the empiric use of antimalarial therapies in children presenting with febrile illness until a malarial blood smear is returned negative; and even then given the high false negative rates of malarial films antimalarial therapies may be continued for a longer duration. Duration of fever correlated significantly with antimicrobial therapies and we suspect in part out of a fear of missing acute malaria or bacillary dysentery. However it is well documented that Rotavirus infection is the major cause of AGE in both developed [13] and developing countries [16] but that the prevalence varies among different regions and countries [17]. Studies in Ghana have shown that Rotavirus is a leading cause of AGE [10, 18] and this fact might argue against the broad use of antimicrobials in the afebrile child presenting with acute onset of diarrhea, vomiting and dehydration. In a number of studies from the African region, enteropathogenic bacteria may represent up to 20% of the causative organisms for AGE [16, 19]. These considerations may contribute to our experience of a high use of antimicrobial therapy for a predominantly viral mediated disease [6]. With lower rates of transmitted malaria being reported in some areas, a more stratified approach to the treatment of acute febrile illness with suspected AGE is being instituted [20]. In the absence of robust screening modalities as stool culture and multiplex testing for gastrointestinal pathogens the current treatment strategy of broad spectrum antimicrobials will likely persist. This strategy is reflected in our findings where 95% of children received at least one antibiotic and 75% at least two or more. Additionally, 84% of the patients received antimalarial therapy. Of the patients who were not tested for malaria 71% received antimalarial therapy and of those who tested negative for malaria parasites 80% received antimalarial treatment. Unfortunately, it is not clear from the clinical documentation what the medical decision making was for those 20% of patients who did not receive antimalarial therapies as this could inform the development of more focused treatment protocols. 95% of our patients received antimicrobial therapies for AGE even though the overall prevalence of bacillary dysentery is low compared to viral mediated AGE in this region [18-20]. In the United States and Europe clinical guidelines for the management of AGE do not include routine testing for microorganism unless in the presence of bloody stools, prolonged illness or recent travel [2, 21]. In addition, the routine use of antimicrobials is not routinely recommended as it is known that antibiotic therapy can worsen the presentation of hemolytic uremic syndrome and prolong diarrhea duration. In our region of Northern Ghana, clinicians wishing to limit their resource utilization may benefit from focusing diagnostic testing on patients with prolonged fever, which may help limit the use of antibiotics and antimalarials in this population. Establishing a clear diagnosis as viral in these patients may also allow the clinician to feel comfortable discharging a patient with continued fevers, thus reducing hospital LOS overall.

Degree of dehydration was associated with prolonged LOS and use of IVF in our study. 75% of children were documented to have no dehydration at the time of admission, yet 70% of this group received intravenous fluids. This could reflect poor assessment or documentation of initial hydration status or an inappropriate use of IVF. Our findings do suggest that the degree of dehydration drives resource utilization, so appropriate assessment of hydration status is crucial for appropriate management in a resource limited setting. Adherence to the clinical dehydration scale validated by Goldman *et al*. [22] for each patient with AGE could help with the appropriate use of limited resources (e.g., IVF) in this patient population. The Ghana Standard Treatment Guidelines provide a table for documenting the degree of dehydration and should be routinely used for the clinical assessment of the child presenting with AGE [13]. In agreement with clinical guidelines ORS was offered to 83% of patients. Overall 76% of all patients received at least one type of IVF support. It is known that physicians tend to overuse intravenous fluids in the management of AGE in spite of the known advantages of using ORS [23]. Zinc supplementation has been demonstrated to decrease length of symptoms and promote more rapid recovery in acute gastroenteritis [24]. We found that 84% of our patients received oral zinc supplementation demonstrating high compliance with WHO and UNICEF recommendations.

**Limitations:** there were several limitations to this study. Its retrospective nature limits the data we were able to collect and thereby our understanding of the medical decision making. The lack of robust literature about expected LOS for AGE admissions in West Africa necessitated our use of a posthoc definition for prolonged LOS within our cohort, although our mean LOS of 4 days correlated well with the published study we were able to identify. Many of our predictors were based on subjective clinical findings as recorded in the chart, with no standardized assessment at the time of admission. Perhaps the largest limitation is the lack of diagnostic specificity. We did not routinely test for stool pathogens, with stool cultures obtained on less than 3% of samples and direct stool examination on less than 6%. No viral testing was performed, so the true prevalence of any disease etiology remains unknown. This study was performed at a single hospital in Northern Ghana, which limits our cohort size and external validity.

## Conclusion

In this single site retrospective chart review of pediatric AGE in Northern Ghana, we found that fever duration in pediatric patients hospitalized with AGE was associated with multiple measures of resource utilization, including hospital LOS and use of antimalarials, antibiotics and IVF. By contrast, degree of dehydration was associated only with hospital LOS and use of IVF. These findings may assist clinicians in focusing their diagnostic testing and treatment on patients requiring high use of resources.

### What is known about this topic

- Acute gastroenteritis is a common presenting illness associated with high mortality and morbidity in hospitalized children;
- Guidelines exist for the optimal management of AGE but there is wide variability in the use and adherence to guidelines.

### What this study adds

- Dehydration and duration of symptoms prior to presentation predict prolonged hospital LOS in young children with AGE in Northern Ghana. Encouraging earlier assessment and intervention during the course of illness may help prevent prolonged hospitalization;
- Increasing duration of fever was associated with increased utilization of antibiotics and antimalarials. Targeted diagnostic testing for children with prolonged fever may allow decreased use of these drugs in patients hospitalized for AGE.

## Competing interests

The authors declare no competing interests.
